# Fear as a Cause of Death

**Published:** 1885-07

**Authors:** 


### Fear as a Cause of Death.

The relation of so-called mental and moral processes to death and life are mostly unknown. Illustrations constantly occur in which no solution can be given of the real cause of death in harmony with demonstrated physiological or pathological facts. In the *Medical Press and Circular*, Aug. 13th, the following cases are given and vouched for by reliable authority :

Two young girls were at dinner at their home in Marseilles, when they were told that a special friend of theirs had died the previous night of cholera. At once they became very nervous and left the table percipitately, ordered a cab and told the driver to take them, as fast as possible, to the town of Aix, some distance from Marseilles. When the cab got outside the city the coachman looked through the window to ask the address of the place to which he was to go. He saw one of the girls in convulsions and the other utterly unconscious. In his turn the driver got frightened, abandoned the cab and ran about like a mad man When the police, who were sent for, arrived and opened the cab they found one girl dead and the other dying. A little way up the road they found the coachman lying on his face, dead.

